# iRSVPred: A Web Server for Artificial Intelligence Based Prediction of Major Basmati Paddy Seed Varieties

**DOI:** 10.3389/fpls.2019.01791

**Published:** 2020-02-25

**Authors:** Arun Sharma, Deepshikha Satish, Sushmita Sharma, Dinesh Gupta

**Affiliations:** Translational Bioinformatics Group, International Centre for Genetic Engineering and Biotechnology, Aruna Asaf Ali Marg, New Delhi, India

**Keywords:** basmati, deep learning, images based classification, artificial intelligence, rice variety prediction, variety prediction server

## Abstract

The purity of seeds is the most important factor in agriculture that determines crop yield, price, and quality. Rice is a major staple food consumed in different forms globally. The identification of high yielding and good quality paddy seeds is a challenging job and mainly dependent on expensive molecular techniques. The practical and day-to-day usage of the molecular-laboratory based techniques are very costly and time-consuming, and involves several logistical issues too. Moreover, such techniques are not easily accessible to paddy farmers. Thus, there is an unmet need to develop alternative, easily accessible and rapid methods for correct identification of paddy seed varieties, especially of commercial importance. We have developed iRSVPred, deep learning based on seed images, for the identification and differentiation of ten major varieties of basmati rice namely, Pusa basmati 1121 (1121), Pusa basmati 1509 (1509), Pusa basmati 1637 (1637), salt-tolerant basmati rice variety CSR 30 (CSR-30), Dehradoon basmati Type-3 (DHBT-3), Pusa Basmati-1 (PB-1), Pusa Basmati-6 (PB-6), Basmati -370 (BAS-370), Pusa Basmati 1718 (1718) and Pusa Basmati 1728 (1728). The method has an overall accuracy of 100% and 97% on the training set (total 61,632 images) and internal validation set (total 15,408 images), respectively. Furthermore, accuracies of greater than or equal to 80% have been achieved for all the ten varieties on the external validation dataset (642 images) used in the study. The iRSVPred web-server is freely available at http://14.139.62.220/rice/.

## Introduction

Rice is a major staple food of the world, however, a few varieties of rice are treated as luxury rather than food. Basmati is one of such rice variety which can turn food into affluence. It has been the pride of the Indian subcontinent region for ages. Many countries claim some of their domestically grown rice varieties as basmati but the original basmati is geographically exclusive to a few districts of India and Pakistan (Rai, 2001; Mishra, 2018). Due to this reason, the European Commission (EC) allowed only long grain aromatic rice from India and Pakistan to be packed and sold as basmati in the traditional markets of Eropean Union (EU). In the financial year 2018-19, Indian basmati accounted for over 90% of the overseas basmati rice market, while rest was exported by Pakistan (**Table S1** and **Figure S1**) (India Export Statistics, 2019; Pakistani Rice: Second to all, 2019). The extent of appreciation that basmati has received all around the world can be estimated by the fact that India alone has exported 44,14,562.21 Metric Ton (MT) of basmati rice to 155 countries of the world (estimated worth ₹ 32,804.19 crores or 4,722.46 million US$) during the year 2018-19 (**Figure S2**) (Ghosal, 2019). The characteristic features of basmati rice which make it world-class are attributed to agro-climatic conditions of the specific geographical area, harvesting methods, as well as processing and aging (**Table S2**). These types of characteristics can help in the identification of milled (precooked or cooked) rice varieties but such characteristics are not clear for basmati paddy seeds (unprocessed/crude form of rice). However, to maintain the demand in the international market, the authenticity of basmati has to be maintained at both producer (farmers) as well as exporter levels. Owing to the highly similar shape and size, it is extremely difficult to differentiate between various basmati seed varieties visually. Thus, the purity identification of paddy seeds is a very challenging task if done manually. It also leads to different types of impediments at various levels. At the producer level, adulterated seeds result in the growth of plants of different heights or stages possessing different time-frames of disease occurrence, ripening, harvesting, etc., making the life of farmers very difficult at different stages of plant growth. Additionally, impurities in grown seeds lead to a reduction in quality grain production, crop price and reliability of exporters, importers and consumers on their producers (Chaugule and Mali, 2016).

According to a survey conducted by The Ministry of Environment, Forests and Climate Change (MoEF and CC), Govt of India, 100% of farmers were depending on the tag on seed bag in the Aligarh market area of Uttar Pradesh province of India (Report on Identity Preservation of Basmati Rice at Various Stages in the Rice Supply Chain). Furthermore, the same survey suggested that in Haryana (India) province too, a large number of farmers (almost 20%) were dependant on the purchased seeds tags. The adulteration in seeds at any level may lead to the supply of impure seeds to farmers.

To overcome the problem of variety identification in India, the Agricultural and Processed Food Products Export Development Authority (APEDA) at the Centre for DNA Fingerprinting and Diagnostics (CDFD, India) and Centre for Basmati DNA analysis (Department of Biotechnology, Government of India), have developed a protocol for DNA Fingerprinting for varietal identification of basmati rice. However, this facility is not appealing to the farmers and exporters of India and Pakistan, owing to the high cost per sample analysis and far location of the DNA analysis centre.

Thus, an easily accessible, easy to use and cost-effective identification of paddy seed varieties is an unmet need for the paddy farmers. In the past, *in-silico* studies have highlighted the importance of morphological characteristics in the classification of various paddy seed varieties. For example, Huang et al. used image segmentation and shape features of paddy seeds (Lemma, plea, glume, and chaff-tip) to classify three varieties (namely Taikong 9, Tainan 11 and Taikong 14) with an average accuracy of 95.21% (Huang and Chien, 2017). Kuo et al. used microscopic images of 30 rice varieties (quantified from 1500 grains) and calculated four different types of traits *viz.* morphological traits (12 traits), color traits (9 traits), textural traits (7 traits) and Fourier descriptors (20 descriptors). The overall accuracy was 89.1% (Kuo et al., 2016). Ruslan et al. have used four morphological features i.e., seed length, width, aspect ratio, and rectangular aspect ratio, for the classification of four local paddy seed varieties namely MR219, MR220, MR263 and MR 269 (Ruslan et al., 2018).

In another study, Chaugule et al. demonstrated the utility of Horizontal-Vertical and Front-Rear angles in the classification of four paddy grains namely Karjat-6, Karjat-2, Ratnagiri-4 and Ratnagiri-24. The classification accuracy of 95.2% achieved using Colour–Shape–Texture was lower than the accuracy of 97.6% achieved using angle features. Moreover, there are several challenges in the calculation of morphological features (Chaugule and Mali, 2016). The authors later on proposed new features based on scaling, rotation and translation of the invariant to classify the paddy seeds with an accuracy of 98.8% (Chaugule and Mali, 2017). Asif et al. used morphological features *viz.* eccentricity, major axis length, minor axis length, perimeter, area and size of the grains, for the quality analysis of six varieties of rice. An automated system is reported to extract morphological features from image for classification and quality analysis but graphical user-interface (GUI) is not provided for end-users to use the developed system (Asif et al., 2018). Apart from above-mentioned studies, many studies also have highlighted the importance of morphological and angle related characteristics or features in the classification of different paddy seeds varieties (Liu Z., et al., 2005; Cervantes et al., 2016; Tekalign and Dileep, 2017; Aznan et al., 2017; Sethy and Chatterjee, 2018) but none of these provide urgently required GUI (except the one (Liu Y., et al., 2005), that too has GUI developed in a non-English language) for end-users nor classifies ten varieties used in the present study. The majority of algorithms made use of the flatbed scanner for the acquisition of images followed by pre-processing, segmentation, edge detection and feature calculation using different image processing software (Punthumast et al., 2012; Tan et al., 2019). The calculated feature values have been further used as input to neural networks for the development of paddy seed variety classification models. Thus, the calculation of features required for classification (in case of previous studies) is a daunting task and out of reach of non-scientific persons i.e., farmers, rice consumers, importers and exporters. Further, a few scientific studies developed methods for classification and grading of milled basmati rice but none of them was dedicated to basmati variety prediction (Gujjar and Siddappa, 2013; Kaur and Singh, 2013; Prajapati and Patel, 2013; Mahajan, 2014).

Keeping in view the above mentioned scientific studies and their short-comings, we have developed an easy to use method for classification of ten major varieties of basmati (global high economic importance and visually highly similar). A large number of images have been used in training and testing the deep neural network models (possess automatic features calculation and selection capability) to classify the basmati paddy seeds with high accuracy. Best performing deep neural network-based models have been used to develop a freely accessible user-friendly web-server “iRSVPred”. It is the first of its own kind, time-efficient, publicly available web-server for basmati seeds variety classification (based on image classification using deep learning). The current version of the web-server requires submission of seeds image in recommended conditions and format (**Table S3**). The iRSVPred may perform poorly for randomly captured images i.e., not in the recommended format. The iRSVPred outputs the predictions of the input image to be of basmati varieties, with corresponding probabilities. We believe that iRSVPred can help basmati exporters, importers, growers and scientists through its user-friendly web-interface.

## Materials and Methods

**Figure 1:**
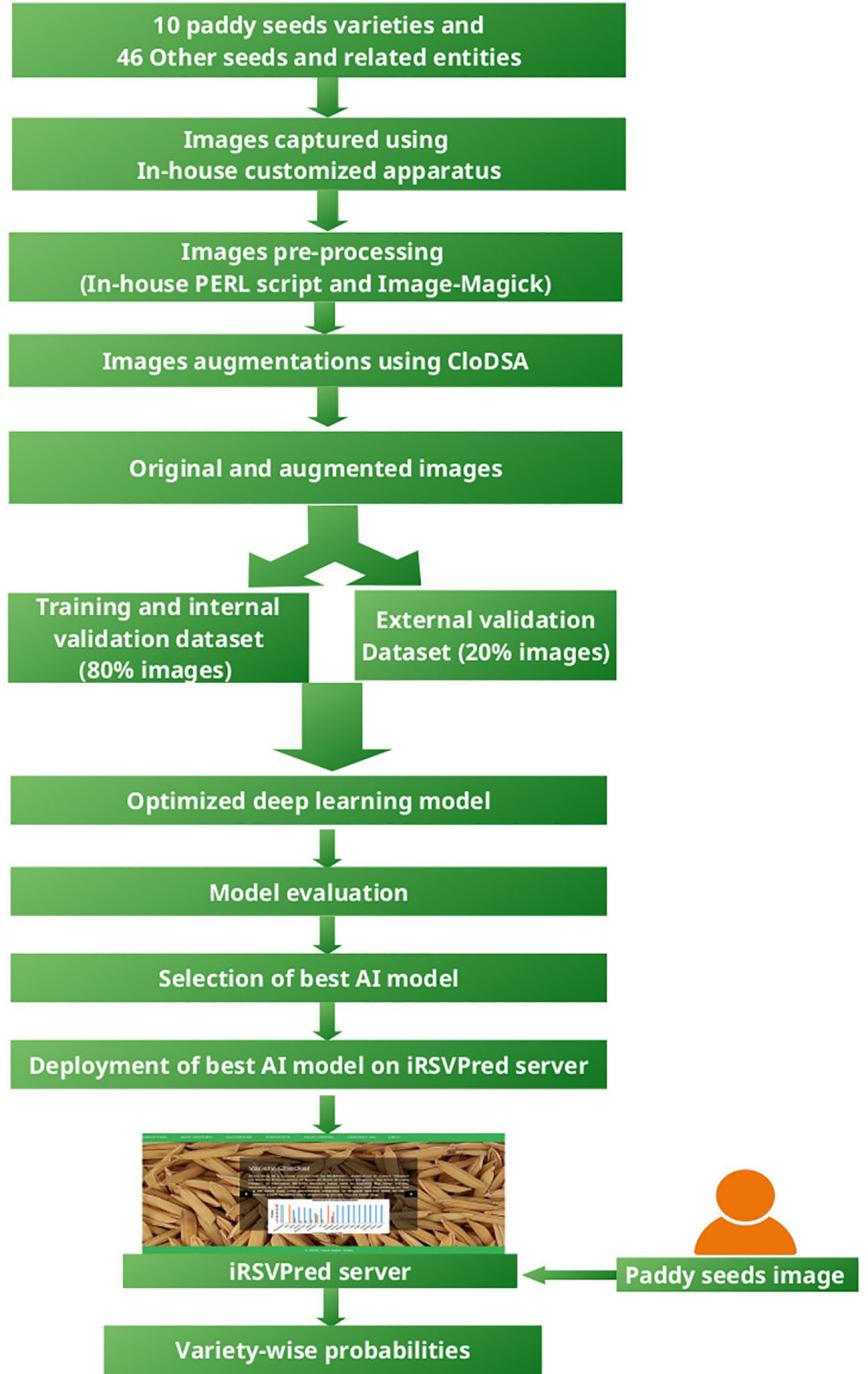
Flowchart for the methodology used for the development of iRSVPred web-server.

**Figure 1** depicts the overall work-flow of iRSVPred, it summarizes the strategy used for the development of artificial intelligence based prediction models used for the iRSVPred web-server.

### Basmati Paddy Seeds Source

A total of ten different paddy variety seed (**Table S4**) samples were collected from the Indian Agricultural Research Institute (IARI), New Delhi, India. Of these ten varieties, five variety of seeds i.e., 1121, 1509, 1637, 1718 and 1728, were collected from Seeds Production Unit (https://www.iari.res.in/index.php?option=com_content&view=article&id=1437&Itemid=1240), while remaining five variety of seeds i.e., BAS-370, CSR 30, Type-3/Dehraduni Basmati, PB-1 and PB-6) were provided by Genetics Department, IARI, New Delhi, India (https://www.iari.res.in/index.php?option=com_content&view=article&id=307&Itemid=1223). The first version of iRSVPred encompasses 10 out of 32 notified basmati seed varieties (**Table S4**).

### Images of Other Seeds, Related Entities and Mixed Paddy Varieties

Of the 10 rice varieties used in iRSVPred, two paddy varieties (1121 and 1509) were mixed to consider them as other seeds and related entities type. Moreover, a number of other seeds and related entities available in the household were also used to capture images of other seeds and related entities category.

### Techniques Used in Generation of Paddy Seed Images

All the images used in the present study were captured manually using an apparatus, developed *in-house*. The dimensions related details of customized apparatus are given in **Table S3**. A five-megapixel camera (5MP) (Micromax Canvas TAB P802) was used to capture all the images used in the training and validation of AI-based prediction models. Originally, the camera captured RGB coloured images of size 2560 × 1920 pixels in the JPEG format. Aperture and focal length of the camera used were f/2.8 and 3.5, respectively. The type of metering mode was centre-weighted average. All the images were taken in standard condition, using customized apparatus. Images were processed using an *in-house* PERL script.

In order to focus on seeds image only, the boundaries of well comprising blank space were cropped using PERL scripts and ImageMagick software (ImageMagick 6.7.8-9). The modified images of size (1450 X 1450) have finally been used for training and validation of the prediction models.

### Augmentation of Modified Paddy Seeds Images

In order to incorporate the variabilities (likely to be incorporated in standard images submitted on iRSVPred web server by users) and increase the dataset size, CloDSA library was used to generate 29 different types of augmentations (**Table S5**) (Casado-García et al., 2019).

### Hardware and Technologies Used for Developing AI-Models and the Web Interface

A workstation with Intel (R) Xeon (R) Gold 6148 CPU @2.40 GZ processors (80 CPUs, 2 NUMA Nodes), 256 GB RAM and CentOS- 7.6.1810 (Core) was used for training and testing the AI models. Originally, the deep learning models training and validation Python scripts were downloaded from https://github.com/sankit1/cv-tricks.com/tree/master/Tensorflow-tutorials/tutorial-2-image-classifier and customized according to requirements of the present study. A brief description of customized scripts and neural network architecture is given in subsequent paragraphs.

Python script (Rossum, 2013) were used for the prediction model development, using open-source libraries such as Open CV2 (Bradski, 2000), OS, glob, shuffle, numpy (Van Der Walt et al., 2011), tensorflow (Martín et al., 2016), time, sys and matplotlib (Hunter, 2007. The entire process used for training and validation of deep learning models may be summarized as: loading of input images data into system memory, shuffle, division into training and internal validation datasets, passing of dataset images through three convolution layers, creation of flattening layer, fully connected layers (FCLs) and prediction outcome. Each image from both training and validation dataset was passed through these steps to calculate the validation loss, training and validation accuracy values and final selection of the best performing model. The best performing models were saved for future use. The detailed information about the entire process is given in the subsequent paragraph.

Python’s OS module was used to communicate with CentOS operating system. The module provided functions to access the paddy seed variety images including counting and names of classes i.e., paddy varieties names. The sklearn.utils (Python scikit-learn) module's “shuffle” utility was used to shuffle the images data prior to training and validation preparation. Open CV2 was used to read, resize (128 X 128), and rescale the paddy varieties images, following this the NumPy library functions helped in the process of converting the image data into numerical matrix which helped in final preparation of training and internal validation dataset. Hence, the NumPy library was used to load the datasets into the system's memory during the training and internal validation of deep learning models. The usage of Tensorflow was started with the creation of convolutional layers and ended with saving the best deep learning model (with the highest accuracy and lowest loss for both, training as well as validation set images). Three convolutional layers *viz.* Conv1, Conv2 and Conv3 were created with 32, 32 and 64 filters, respectively. The filter size of 3x3 was applied, accompanied with the number of channel value of 3 (in case of RGB images) for each conventional layer. Every conventional layer was followed by the application of “Max Pooling” (to reduce the input image's dimensions while keeping the important feature values after each convolution applied) and rectified linear unit (ReLU) as an activation function. It was required to pass useful information from one hidden layer to the next hidden layers in order to identify the features varying among the ten paddy varieties, along with other seeds and related entities. After applying the conventional layers, a flatten layer was created to convert the feature values into one dimension. The creation of flatten layer was followed by the building of two fully connected layers (FCLs) and the application of “Softmax” at the last FCL to get output prediction (in terms of probability) for an image used during the training of deep learning model. The “Adam” optimizer with a learning rate value of 1e-4 and cross-entropy functions was also used to minimize loss value and maximize prediction accuracy (%) value. Thus, the Adam optimizer helped in the optimization of iRSVPred prediction models in time and resource-limited facilities. Finally, the best performing models were saved and rendered available for use *via* a GUI or web-interface, using PHP (PHP 7.1.28), CSS (Wium Lie, 1996), and AJAX (Garrett, 2005) open-source technologies.

### AI-Based Prediction Models Development

Thirty-two AI models (**Table S6**) were developed using different combinations of images generated through the augmentations generated using the CloDSA library. Three different approaches were used for developing the AI-based basmati variety prediction models. Firstly, only original images (a total of 2055 and 513 images were used for training and internally validating the models, respectively) were used for model building (**Figures S3.1** and **S3.2**). Secondly, the original images and individual augmentation were used (original images and one augmentation at a time while training the AI models) to develop single augmentation models (**Figure S4**). Thus, 29 different types of single augmentation models were generated corresponding to 29 different types of augmented images based models. Furthermore, multiple augmentations and original image based models were developed and evaluated, separately. To ensure the best possible performance of models, the two latter models were developed on 251 and 502 epochs, upon which accuracy (for training and internal validation sets) was consistently maintained at the number of epochs. All these models were developed using image size of 128 x 128, batch size (BS) of 256, 3 number of channels (NC), 3 convolutional layers while the number of epochs and iterations were varied. For the models based on the original images, combination model of the original images and individual augmentation images, 250 epochs were used to train while original images plus multiple augmentation model-I (MAM-I) and multiple augmentation model-II (MAM-II) were trained on both 251 and 502 epochs, respectively. The time taken to train the models with 251 and 502 epochs was ~24 hours (CPU Mode) and ~41 hours (CPU Mode), respectively.

The training dataset was further internally spilt into an 80% training dataset and a 20% internal validation dataset. **Figures S4**, **S5** and **S6** show the methodology used for validation of external validation set images on three different types of models. The results obtained after training and validating these models are given subsequently.

**Figure 2:**
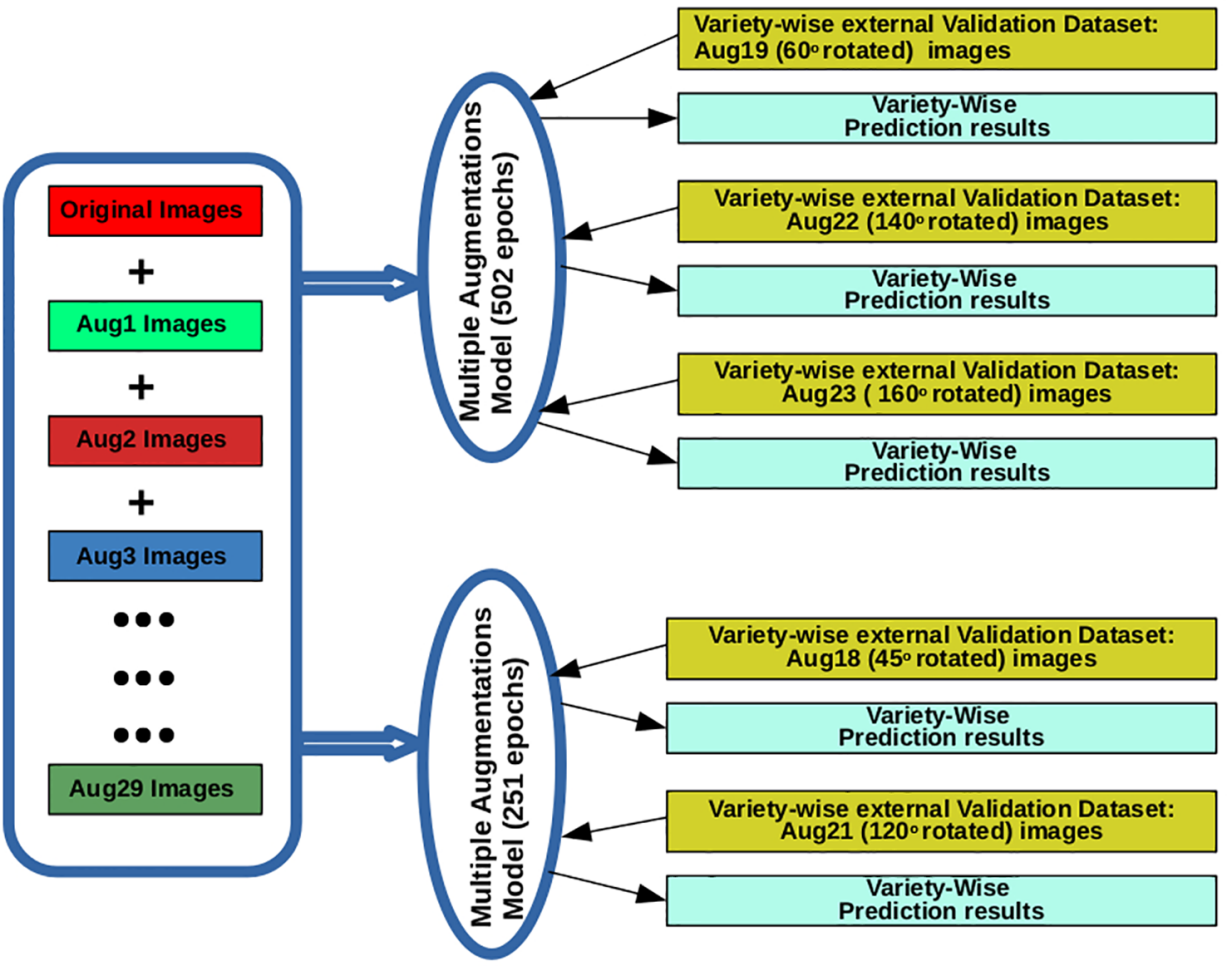
The training and validation of AI- based prediction models (used for the iRSVPred web-server), along with the types of images used.

MAM-I and MAM-II models were tested on external validation set containing all augmentation types for each of the varieties. Finally, augmentation types, i.e., rotate 45° (Aug18), rotate 120° (Aug21) and rotate 60° (Aug19), rotate 140° (Aug22), rotate 160° (Aug23) were found to provide higher prediction accuracies (%) for at least 8 varieties when tested on 251 epoch and 502 epoch model, respectively. These two models with the above mentioned five types of augmentations were selected to be deployed on iRSVPred server (**Table 2** and **Figure 2**). A complete list of expanded forms of abbreviations used for augmentations are given in the **Table S5**.

The iRSVPred server (freely available at http://14.139.62.220/rice/) is hosted on a workstation having Quad-Core AMD Opteron (tm) Processor 2384 with 8 CPUs (2 NUMA nodes) and 32 GB RAM, with Ubuntu 18.04.2 LTS OS.

### Availability of Source Codes

The codes used for development and validation of AI-based prediction models, including sample images, developed models and scripts used for augmentation of the original images, have been uploaded on GitHub (https://github.com/arunsharma8osdd/iRSVPred).

### Statistical Evaluation of Models

The percentage accuracies was used to evaluate the performance of trained models on internal and external validation datasets. The formula used for accuracy (%) calculation is as given below:

(1) $$Accuracy\;(\%)\;for\;variety\;i=\frac{Correctly\;predicted\;images\;(variety\;i)}{Total\;number\;of\;images\;(variety\;i)}*100$$

where the values of i are the basmati paddy varieties used for developing the AI-based prediction models i.e., 1121, 1728, 1509, etc.

### External Validation Datasets

For external validation of trained AI models, we used 642 original images, 1284 original images with individual augmentations and 19260 original images with multiple augmentations. The validation images were not used for the training and internal validation of the AI-based prediction models.

## Results

### Original Images Used for Training and Validation of AI-Models

A total of 3210 original images were captured (**Table S7**, **Figure S3.1**). Out of these 3210 images, 710 images belong to “other seeds and related entities” class.

The “Other seeds and related entities” class comprises 460 images of 46 different seeds commonly available while 250 images were a mixture of 1121 and 1509 varieties (**Figure S7**). The “Other seeds and related entities” dataset was used in order to eliminate the possibility of accidental input of images other than paddy seed or other grains/cereal images to the web server.

### Augmented Images Used for Training and Validation of AI-Models

**Figure 3:**
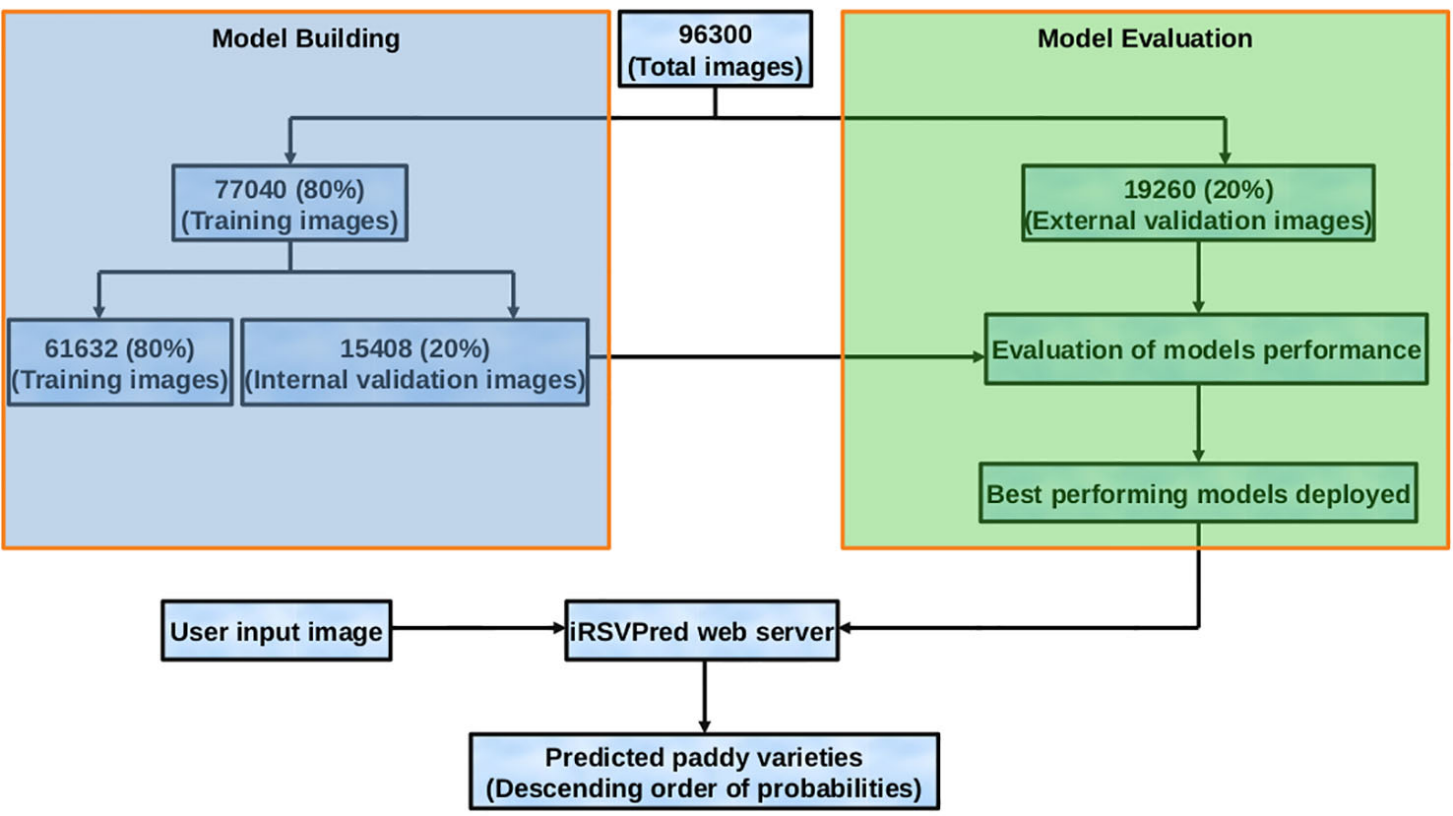
Flow diagram representing dataset preparation, model building and evaluation, along with usage of the best prediction models (on iRSVPred web-server).

In order to artificially enhance and enrich the training dataset, 93090 images (3210 x 29) were generated using CloDSA augmentation. Hence, the final image training dataset consists of original image dataset as well as the augmented images, making the number of dataset images to be 96300. Out of this large dataset, 80% (77048 images) were used for model generation and internal validation, while rest of the 20% (19260 images) were used as external validation data set (**Figure 3**, **Figure S8**). These validation images were not used in model generation, and served as an independent test dataset.

### Overall Performance of Prediction Models on Training and Internal Validation Dataset

Thirty two models were trained and internally validated on images of ten paddy seed varieties and images of 46 different seeds and related entities (details of model generation discussed in the methods section). The training and internal validation-set accuracies, along with validation loss values recorded during training of prediction models are given in **Table S6**.

The **Table S6** is the testament to the fact that, the models based on ~62,000 images performed equally efficient when compared to the lesser (~4000) images based models. A larger and quality training dataset for algorithm training often results in better trained models with accurate results on validation/real-life datasets (Kadam and Vaidya, 2020). Hence, we selected the two models, trained on 61632 images only, as our final prediction models. Thus, these two models have been used on web-server for prediction i.e., “Multiple augmentation model-I (MAM-I)” and “Multiple augmentation model-II (MAM-II)”, were trained on 251 and 502 epoch, respectively with training accuracies of 98% and 100%, respectively (**Table 1**).

**Table 1:** Training and internal validation set accuracies accompanied with validation loss values for paddy seeds variety prediction models developed using original images and different types of augmented images.

Sr. No.	Augmentation type	No. of training set images	No. of internal validation set images	Training accuracy (%)	Validation accuracy (%)	Validation loss
1	Multiple augmentation model-I (MAM-I) (251 Epochs)	61632	15408	98.0	93.4	0.239
2	Multiple augmentation model-II (MAM-II) (502 Epochs)	61632	15408	100.0	97.7	0.155

In order to get the most efficient and reliable model, a stringent model selection criteria was chosen. Only those models were selected, for which an accuracy of greater than or equal to 80% was achieved (on external validation dataset) for at least 8 paddy varieties. A number of different types of external validation images were chosen to test the prediction efficiency of AI-based prediction models, whose results are given in **Tables S8–S13.2**. The best performing models with their results are given in **Table 2**.

**Table 2:** Multiple augmentation models (251 and 502 Epochs respectively) performance on external validation dataset.

Sr. No.	Variety/other seeds and related entities	No. of images used for validation	Accuracy (%) for images using MAM-I model		Accuracy (%) for images using MAM-II model		
			**45**° **rotated**	**120**° **rotated**	**60**° **rotated**	**140**° **rotated**	**160**° **rotated**
1	1121	50	62	64	86	82	80
2	1509	50	96	98	96	98	98
3	1637	50	92	90	90	100	98
4	1718	50	60	60	84	84	82
5	1728	50	86	82	56	68	56
6	BAS 370	50	98	98	100	100	100
7	CSR 30	50	96	98	96	90	92
8	DHBT 3	50	100	100	100	100	100
9	PB 1	50	86	84	92	84	98
10	PB 6	50	80	80	64	68	66
11	Other seed and related entities	142	100	100	99.3	100	100

### Performance of Prediction Models (Variety-Wise) on External Validation-Dataset

As evident from **Table 2**, for MAM-I trained on 251 epochs, the highest accuracy of 100% was achieved using external validation dataset images for DHBT-3. Highest accuracy of 98% was achieved for 1509, BAS-370 and CSR-30 varieties. The six varieties 1637, 1728, PB-1, PB-6, 1121 and 1718 were predicted with highest accuracies of 92%, 86%, 86%, 80%, 64% and 60%, respectively.

Further, for MAM-II trained on 502 epochs, the highest accuracy of 100% was achieved using external validation dataset images for varieties 1637, BAS-370 and DHBT-3 followed by accuracy of 98% for 1509 and PB-1 varieties. The five varieties CSR-30, 1121, 1718, 1728 and PB-6 were predicted with accuracies of 96%, 86%, 84%, 68% and 68%, respectively.

**Figure 4:**
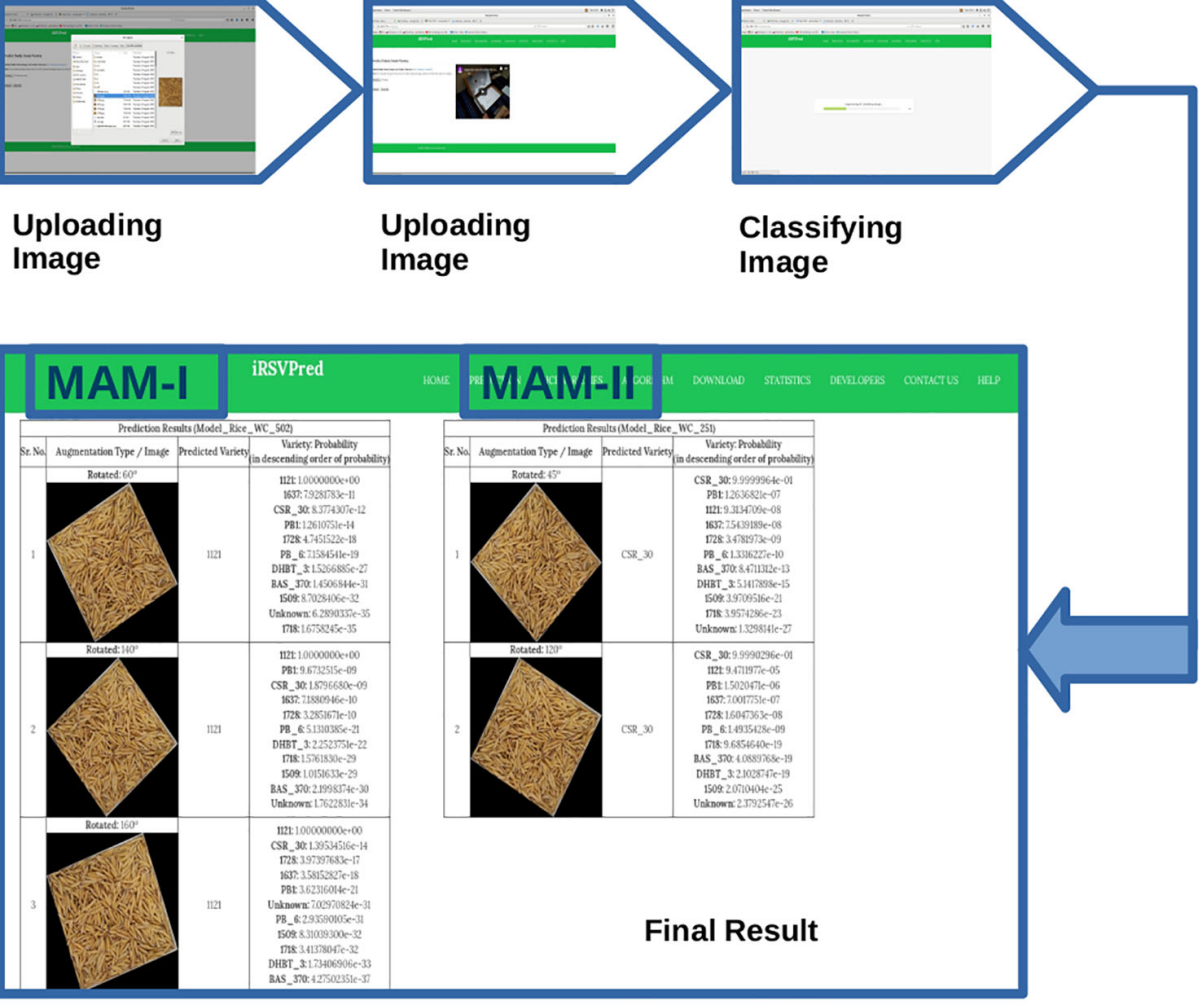
Screenshots showing step-by-step usage of iRSVPred web-server.

Thus, MAM-I (251 epochs) showed poor performance for, 1121 and 1718 varieties, while MAM-II (502 epochs) showed lower performance for 1728 and PB-6 varieties. In order to overcome the poor performance, iRSVPred server has been designed such that simultaneous prediction results by both the models are shown for user submitted query images (**Figure 4**).

### iRSVPred User Interface

A user-friendly web interface “iRSVPred” has been designed for the easy access and use of the prediction models by users. The Graphical User Interface (GUI) is divided into eight different sections or tabs *viz.* Home, Prediction, Rice Varieties, Algorithm, Statistics, Developers, Help, and Contact Us. A brief introduction to the basmati paddy varieties and its importance is available on the Home page. Additionally, objectives and need of iRSVPred is also addressed.

The “Prediction” tab of iRSVPred is the interface to the main function of the server i.e. prediction of ten major paddy varieties using two selected prediction models. On this web-page, one can submit query image of rice seeds (example images and videos are available on website to help users). The recommended specifications to click standard images are also given in the **Table S3**). The query will give a non-ambiguous model-wise and probability-wise (in descending order of probabilities) list of different predicted varieties along with augmented images (**Figure 4**).

## Discussion

Previous AI-based paddy variety prediction models made use of object analysis method for AI model training. In contrast to this iRSVPred is the first ever AI-based web-server to predict 10 major basmati variety seeds on the basis of seed images (**Table S4**). The image-based analysis is comparatively advantageous in comparison to object analysis as the latter, which is not able to consider variability among seeds of the same species (simultaneously), that may have arisen due to varied ecological conditions and other factors. A number of different augmentations have been applied to increase the number of images and their diversity.

Despite having high classification accuracy, one of the major limitation of previous studies is the requirement to calculate morphological or other characteristics manually or using a number of complex apparatuses. The former calculations are tedious and time-consuming while latter options have limited availability. Also, the chances of incorporation of errors while the calculation of these feature values are a matter of grave concern as this may lead to false predictions. Thus, these studies are away from the reach of real users such as farmers or rice consumers. To the best of our knowledge, iRSVPred is the first ever method which is an images-based paddy seeds classification server, wherein automatic feature extraction takes place (in back-end through deep learning models).

In the present study, knowingly, a medium resolution camera (just 5MP) has been used for capturing paddy seed pictures so that prediction accuracy could be maintained even after getting submission of poor quality images from iRSVPred users. The image capturing apparatus used in the present study can easily be prepared because of its simple methodology of preparation and low cost of raw materials. The approximate cost for building this apparatus may be ₹ 200 (2.83 USD, including the cost of two LED bulbs and a cardboard box). Once prepared, the image capturing box can be used a number of times and its constituents may be reused for other purposes later. Thus, iRSVPred server is a proof of concept to show that AI models trained with representative paddy seed images of different classes offer an alternative and efficient prediction method.

During validation of the models, model training with rotated images conferred higher prediction accuracies, hence, query images uploaded by users (on iRSVPred web-server) are also rotated through five different angles i.e., 45°, 120° (for 251 epochs based model) and 60°, 140°, 160° (for 502 epochs based model), for getting highly accurate prediction results. The selection of models has been done in such a way that one model performing poorly for two varieties is overcome by another model's good performance for those varieties and vice-versa. In order to prevent the submission or prediction of non-relevant and adulterated paddy images, other seeds and related entity image datasets were prepared and used during model building and validation. As the models have been trained on images captured in standard conditions, therefore, it is recommended to capture new images in standard conditions as defined in **Table S3**.

### Relevance and Applications of the Present Study

It is a daunting task to physically identify the exact paddy variety. Many wet-lab, chemical-based methods are available to distinguish between the basmati paddy varieties at the genetic level. But these methods are time-consuming and expensive. iRSVPred can serve as a cost-effective and efficient method to identify the basmati paddy seed varieties. Though iRSVpred is not a replacement for chemical methods, it is freely accessible and can be used for rapid screening or complementation to the present methods to increase the robustness of results.

### Limitations and the Future Prospects

The present version of iRSVPred encompasses 10 widely used basmati varieties. The future versions of iRSVPred will incorporate all the authentic basmati varieties available in the subcontinent. Secondly, in the current version of iRSVPred, the input pictures are required to be taken in standard conditions, for better accuracy. The future versions of iRSVPred, with larger datasets and improved alogorithms, may be able to perform predictions on free-style/randomly captured paddy images.

## Data Availability Statement

For external validation, dataset has been provided at iRSVPred website itself.

## Author Contributions

AS and DS contributed equally to this work. AS, DG, and DS conceived and designed the study. DS collected the paddy seeds from IARI, New Delhi, India and captured images required for the study. AS and DS developed the AI-based prediction models and collected the results. SS designed the web pages of iRSVPred web-server and AS developed the interface for using AI models by users. DG, AS, and DS drafted the manuscript. All authors wrote and reviewed the manuscript.

## Funding

This work was financially supported by the Department of Biotechnology (DBT), Government of India, grants BT/BI/04/001/2018 and BT/BI/25/066/2012. AS acknowledges DBT Apex Biotechnology Information Centre at International Centre for Genetic Engineering and Biotechnology (ICGEB, India), for financial assistance. DS received fellowship from the Council of Scientific and industrial Research (CSIR, 09/0512(0207)/2016/EMR-1), New Delhi, India.

## Conflict of Interest

The authors declare that the research was conducted in the absence of any commercial or financial relationships that could be construed as a potential conflict of interest.
