# Supplementary material for: iRSVPred: A Web Server for Artificial Intelligence Based Prediction of Major Basmati Paddy Seed Varieties
Source: Front Plant Sci. 2020 Feb 25;10:1791. doi: 10.3389/fpls.2019.01791 (PMC7052261; doi:10.3389/fpls.2019.01791)

**Supplementary Information**

**iRSVPred: A web server for artificial intelligence based prediction of major basmati paddy seed varieties**

**Arun Sharma**^#^**, Deepshikha Satish**^#^**, Sushmita Sharma, Dinesh Gupta^*^**

**Translational Bioinformatics Group, International Centre for Genetic Engineering and Biotechnology, Aruna Asaf Ali Marg, New Delhi, India**

***To whom the correspondence should be addressed**

Dr. Dinesh Gupta,

Group Leader, Translational Bioinformatics Group,

International Centre for Genetic Engineering and Biotechnology,

Aruna Asaf Ali Marg, New Delhi, India.

Pin- 110067

Email: dinesh@icgeb.res.in

Phone: 011 2674 1358 (403)

**^#^Equal contribution**

**Table S1: The export of Basmati rice from India during the last three years:**

| **2016-17** | | | **2017-18** | | | **2018-19** | | |
| --- | --- | --- | --- | --- | --- | --- | --- | --- |
| **Qty-MTs** | **Val-₹ cr** | **Val-$ m** | **Qty-MTs** | **Val-₹ cr** | **Val-$ m** | **Qty-MTs** | **Val-₹ cr** | **Val-$ m** |
| 39,85,195 | 21,513 | 3,217 | 40,56,759 | 26,870 | 4,169 | 44,14,562 | 32,804.19 | 4,722.46 |

**Source:** The Directorate General of Commercial Intelligence and Statistics (DGCI & S), Kolkata, under the Ministry of Commerce, Government of India

**Table S2: Primary basmati quality characteristics**

Source of table 3: The Department of Agriculture, Cooperation and Farmers’ welfare, Govt. of India

** The grain sample for analyses will necessarily have to be 'aged' for three months under protected conditions at normal room temperature as milled kernel.

* As per standardized protocol (Directorate of Rice Research, Hyderabad)'

| **S. No.** | **Parameter**** | **Value** |
| --- | --- | --- |
| 1 | Minimum average precooked milled rice length (mm) | 6.61 |
| 2 | Average precooked milled rice breadth (mm) | <=2.00 |
| 3 | Minimum length/breadth ratio of precooked milled rice (L/B Ratio) | 3.50 |
| 4 | Minimum average cooked rice length (mm) | 12.00 |
| 5 | Minimum cooked rice length/precooked rice length ratio or Minimum elongation ratio | 1.70 |
| 6 | Average volume expansion ratio | >3.50 |
| 7 | Aroma | Present (Qualitative Sensory Analysis as Panel Test*) |
| 8 | Texture of cooked grain for high integrity (without bursting the surface), non-stickiness, tenderness, good taste and good mouth feel | Present (Qualitative Sensory Analysis as Panel Test*) |

**Table S3: Specification of apparatus.**

| **S. No.** | **Specifications** | **Detail** |
| --- | --- | --- |
| 1 | Dimension of Apparatus | 20cm * 33.5 cm (Breadth * Height) |
| 2 | Background | White |
| 3 | Height from sample | 11 cm |
| 4 | Area of sample well | 8.5cm*8.5cm*1cm |
| 5 | Light Used | 2 Light-emiting diode bulb of power 4 Watt each was used. Light emitted by each bulb was crystal white of luminous flux 370 lumen. Bulbs were positioned diagonally opposite in order to cancel the shadow effect. |

**Table S4: List of 32 basmati varieties notifies by Govt. of India. (Varieties used in iRSVPred are highlighted in bold letters)**

| **S. No.** | **Variety** | **Notification No. & Date** |
| --- | --- | --- |
| 1 | Basmati 217 | 4045 – 24.09.1969 361 (E) – 30.06.1973 |
| 2 | Punjab Basmati 1 (Bauni Basmati) | 596 (E) - 13.08.1984 |
| 3 | Basmati 386 | 647 (E) – 09.09.1997 |
| 4 | Punjab Basmati 2 | 1708 (E) – 26.07.2012 |
| 5 | Punjab Basmati 3 | 3540(E) - 24.11.2016 |
| **6** | **Basmati 370** | **361 (E) – 30.06.1973786 – 02.02.1976** |
| 7 | Haryana Basmati 1 | 793 (E) - 22.11.1991 |
| 8 | Taraori Basmati (HBC 19) | 1(E) – 01.01.1996 |
| **9** | **Type 3 (Dehraduni Basmati)** | **13 – 19.12.1978** |
| 10 | Pant Basmati 1 (IET 21665) | 112(E)- 13.01.2016 |
| 11 | Pant Basmati 2 (IET 21953) | 112(E) - 13.01.2016 |
| 12 | Kasturi | 615 (E) - 06.11.1989 |
| 13 | Mahi Sugandha | 408 (E) - 04.05.1995 |
| **14** | **Basmati CSR 30**  **After amendment** | **1134(E) – 25.11.2001**  **2126 (E) – 10.09.2012** |
| 15 | Malviya Basmati Dhan 10-9 (IET 21669) | 2817 (E) – 19.09.2013 |
| 16 | Ranbir Basmati | 1 (E) - 01.01.1996 |
| 17 | Basmati 564 | 268 (E) – 28.01.2015 |
| **18** | **Pusa Basmati 1** | **615 (E) - 06.11.1989** |
| 19 | Improved Pusa Basmati 1 (Pusa 1460) | 1178 (E) – 20.07.2007 |
| **20** | **Pusa Basmati 1121**  **After amendment** | **1566 (E) – 05.11.2005**  **2547 (E) - 29.10.2008** |
| **21** | **Pusa Basmati 1509 (IET 21960)** | **2817 (E) – 19.09.2013** |
| **22** | **Pusa Basmati 6 (Pusa 1401)** | **733 (E) – 01.04.2010** |
| 23 | Pusa Basmati 1609 | 2680(E)- 01.10.2015 |
| **24** | **Pusa Basmati 1637** | **3540(E) - 24.11.2016** |
| **25** | **Pusa Basmati 1728** | **3540(E) - 24.11.2016** |
| 26 | Vallabh Basmati 22 | 2187 (E) – 27.08.2009 |
| 27 | Vallabh Basmati 21 (IET 19493) | 2817 (E) – 19.09.2013 |
| 28 | Vallabh Basmati 23 | 268 (E) – 28.01.2015 |
| 29 | Vallabh Basmati 24 | 268 (E) – 28.01.2015 |
| **30** | **Pusa Basmati 1718** | **2805(E) - 25.08.2017** |
| 31 | Punjab Basmati 4 | 1379 (E) – 27.03.2018 |
| 32 | Punjab Basmati 5 | 1379 (E) – 27.03.2018 |

**Table S5: Full- form of the abbreviations of Augmentations used in iRSVPred**

| **Sr. No.** | **Augmentation abbreviations** | **Full Form** |
| --- | --- | --- |
| 1 | Aug1 | HSV(Hue, Saturation, and Value) |
| 2 | Aug2 | Gamma Correction |
| 3 | Aug3 | LAB(L* for the lightness from black to white, A* from green to red, and B* from blue to yellow) |
| 4 | Aug4 | Crop 0.5 |
| 5 | Aug5 | Crop 0.7 |
| 6 | Aug6 | Crop 0.9 |
| 7 | Aug7 | Drop out 5 % |
| 8 | Aug8 | Drop out 10 % |
| 9 | Aug9 | Drop out 15 % |
| 10 | Aug10 | Elastic Deformation |
| 11 | Aug11 | Equalize Histogram |
| 12 | Aug12 | Flip Horizontal |
| 13 | Aug13 | Flip Vertical |
| 14 | Aug14 | Flip Horizontal followed by vertical |
| 15 | Aug15 | Invert |
| 16 | Aug16 | Shearing |
| 17 | Aug17 | Sharpen |
| 18 | Aug18 | Rotate 45 degree |
| 19 | Aug19 | Rotate 60 degree |
| 20 | Aug20 | Rotate 90 degree |
| 21 | Aug21 | Rotate 120 degree |
| 22 | Aug22 | Rotate 140 degree |
| 23 | Aug23 | Rotate 160 degree |
| 24 | Aug24 | Resize |
| 25 | Aug25 | Raise Red |
| 26 | Aug26 | Raise Hue |
| 27 | Aug27 | Raise Green |
| 28 | Aug28 | Raise Blue |
| 29 | Aug29 | Median Blur |

**Table S6: Training and internal validation-set accuracies accompanied with validation loss values for paddy seeds variety prediction models developed using original images and different types of augmented images.**

| **Sr. No.** | **Model Type** | **No. of training set images** | **No. of internal validation set images** | **Training**  **Accuracy (%)** | **Validation Accuracy (%)** | **Validation Loss** |
| --- | --- | --- | --- | --- | --- | --- |
| 1 | Original images Model | 2055 | 513 | 99.20 | 88.30 | 0.31 |
| 2 | Original + Crop 0.5 images Model | 4109 | 1027 | 100.00 | 86.30 | 0.496 |
| 3 | Original + Crop 0.7 images Model | 4109 | 1027 | 99.20 | 87.10 | 0.345 |
| 4 | Original + Crop 0.9 images Model | 4109 | 1027 | 95.70 | 85.90 | 0.486 |
| 5 | Original + Dropout 0.05 images Model | 4109 | 1027 | 100.00 | 94.10 | 0.154 |
| 6 | Original + Dropout 0.10 images Model | 4109 | 1027 | 100.00 | 87.90 | 0.349 |
| 7 | Original + Dropout 0.15 images Model | 4109 | 1027 | 100.00 | 91.00 | 0.23 |
| 8 | Original + Elastic Deform images Model | 4109 | 1027 | 100.00 | 96.10 | 0.084 |
| 9 | Original + Equalize Histogram images Model | 4109 | 1027 | 100.00 | 87.90 | 0.385 |
| 10 | Original + Flip horizontal images Model | 4109 | 1027 | 99.60 | 90.60 | 0.307 |
| 11 | Original + Flip vertical images Model | 4109 | 1027 | 99.60 | 92.60 | 0.255 |
| 12 | Original + Flip both images Model | 4109 | 1027 | 99.60 | 89.50 | 0.287 |
| 13 | Original + Gamma correction images Model | 4109 | 1027 | 99.60 | 98.00 | 0.079 |
| 14 | Original + HSV images Model | 4109 | 1027 | 96.50 | 92.60 | 0.229 |
| 15 | Original + Invert images Model | 4109 | 1027 | 100.00 | 89.80 | 0.253 |
| 16 | Original + LAB images Model | 4109 | 1027 | 99.20 | 88.30 | 0.38 |
| 17 | Original + Median blur images Model | 4109 | 1027 | 99.60 | 95.70 | 0.135 |
| 18 | Original + Raise blue images Model | 4109 | 1027 | 100.00 | 96.90 | 0.13 |
| 19 | Original + Raise green images Model | 4109 | 1027 | 100.00 | 98.40 | 0.055 |
| 20 | Original + Raise hue images Model | 4109 | 1027 | 100.00 | 97.30 | 0.147 |
| 21 | Original + Raise red images Model | 4109 | 1027 | 100.00 | 98.00 | 0.079 |
| 22 | Original + Resize images Model | 4109 | 1027 | 100.00 | 98.40 | 0.069 |
| 23 | Original + Rotate 45**^o^** images Model | 4109 | 1027 | 98.40 | 93.00 | 0.241 |
| 24 | Original + Rotate 60**^o^** images Model | 4109 | 1027 | 98.80 | 89.50 | 0.275 |
| 25 | Original + Rotate 90**^o^** images Model | 4109 | 1027 | 98.00 | 90.20 | 0.272 |
| 26 | Original + Rotate 120**^o^** images Model | 4109 | 1027 | 98.80 | 91.40 | 0.287 |
| 27 | Original + Rotate 140**^o^** images Model | 4109 | 1027 | 98.40 | 86.70 | 0.434 |
| 28 | Original + Rotate 160**^o^** images Model | 4109 | 1027 | 98.00 | 92.60 | 0.204 |
| 29 | Original + Sharpen images Model | 4109 | 1027 | 100.00 | 92.60 | 0.203 |
| 30 | Original + Shearing images Model | 4109 | 1027 | 100.00 | 91.00 | 0.266 |
| 31 | **Multiple augmentation Model**  **(251 Epochs)** | **61632** | **15408** | **98.0** | **93.4** | **0.239** |
| 32 | **Multiple augmentation Model**  **(502 Epochs)** | **61632** | **15408** | **100.0** | **97.7** | **0.155** |

**Table S7: Number of original images generated**

| **S. No.** | **Variety** | **Number of Images taken** |
| --- | --- | --- |
| 1 | Basmati 370 | 250 |
| 2 | Type 3 (Dehraduni Basmati) | 250 |
| 3 | Pusa Basmati 1 | 250 |
| 4 | Pusa Basmati 1121 | 250 |
| 5 | Pusa Basmati 6 (Pusa 1401) | 250 |
| 6 | Basmati CSR 30 | 250 |
| 7 | Pusa Basmati 1509 (IET 21960) | 250 |
| 8 | Pusa Basmati 1637 | 250 |
| 9 | Pusa Basmati 1728 | 250 |
| 10 | Pusa Basmati 1718 | 250 |
| 11 | Mix varieties (1121 plus 1509) | 250 |
| 12 | Other seeds and related entities | 460 |
|  | Total Images | 3210 |

**Table S8: External validation set (containing all augmentation types for each variety) accuracies on multiple augmentation model (251 and 502 epochs).**

| **Sr. No.** | **Variety / Other seeds and related entities** | **No. of images used for validation** | **Multiple augmentation Model**  **(251 Epochs)**  **Accuracy (%)** | **Multiple augmentation Model**  **(502 Epochs)**  **Accuracy (%)** |
| --- | --- | --- | --- | --- |
| 1 | 1121 | 1500 | 61.4 | 68.53 |
| 2 | 1509 | 1500 | 85.33 | 88.2 |
| 3 | 1637 | 1500 | 59.07 | 77.47 |
| 4 | 1718 | 1500 | 38.07 | 64.47 |
| 5 | 1728 | 1500 | 64.73 | 55.6 |
| 6 | BAS-370 | 1500 | 89.87 | 96.13 |
| 7 | CSR-30 | 1500 | 87.53 | 65.67 |
| 8 | DHBT-3 | 1500 | 83.73 | 95.4 |
| 9 | PB-1 | 1500 | 78.27 | 83.47 |
| 10 | PB-6 | 1500 | 69.87 | 59.53 |
| 11 | Other seeds and related entities | 4260 | 99.51 | 99.27 |

**Table S9: External validation-set (original images) accuracies on paddy seeds variety prediction multiple augmentation model (251 and 502 epochs).**

| **Sr. No.** | **Variety / Other seeds and related entities** | **No. of images used for validation** | **Multiple augmentation Model**  **(251 Epochs)**  **Accuracy (%)** | **Multiple augmentation Model**  **(502 Epochs)**  **Accuracy (%)** |
| --- | --- | --- | --- | --- |
| 1 | 1121 | 50 | 74 | 72 |
| 2 | 1509 | 50 | 92 | 92 |
| 3 | 1637 | 50 | 76 | 84 |
| 4 | 1718 | 50 | 26 | 62 |
| 5 | 1728 | 50 | 86 | 74 |
| 6 | BAS-370 | 50 | 98 | 100 |
| 7 | CSR-30 | 50 | 86 | 64 |
| 8 | DHBT-3 | 50 | 72 | 98 |
| 9 | PB-1 | 50 | 86 | 90 |
| 10 | PB-6 | 50 | 80 | 64 |
| 11 | Other seeds and related entities | 142 | 100 | 100 |

**Table S10.1: External validation-set (original and augmented images) accuracies on paddy seeds variety prediction multiple augmentation model (251 epochs).**

| **Sr. No.** | **Variety / Other seeds and related entities** | **No. of images used for validation** | **Aug1** | **Aug2** | **Aug3** | **Aug4** | **Aug5** | **Aug6** | **Aug7** | **Aug8** | **Aug9** | **Aug10** | **Aug11** | **Aug12** | **Aug13** | **Aug14** | **Aug15** |
| --- | --- | --- | --- | --- | --- | --- | --- | --- | --- | --- | --- | --- | --- | --- | --- | --- | --- |
| 1 | 1121 | 100 | 73 | 65 | 78 | 63 | 71 | 70 | 55 | 64 | 49 | 81 | 46 | 68 | 71 | 68 | 71 |
| 2 | 1509 | 100 | 85 | 84 | 76 | 90 | 85 | 85 | 80 | 93 | 91 | 95 | 75 | 94 | 93 | 93 | 64 |
| 3 | 1637 | 100 | 68 | 48 | 45 | 68 | 75 | 72 | 48 | 51 | 51 | 46 | 39 | 75 | 77 | 75 | 68 |
| 4 | 1718 | 100 | 25 | 22 | 15 | 32 | 35 | 27 | 20 | 28 | 22 | 27 | 22 | 32 | 44 | 35 | 62 |
| 5 | 1728 | 100 | 73 | 67 | 58 | 78 | 82 | 87 | 67 | 64 | 59 | 53 | 60 | 86 | 84 | 86 | 57 |
| 6 | BAS-370 | 100 | 96 | 90 | 90 | 95 | 96 | 96 | 99 | 99 | 99 | 99 | 82 | 92 | 97 | 84 | 90 |
| 7 | CSR-30 | 100 | 87 | 92 | 79 | 85 | 88 | 91 | 84 | 86 | 90 | 85 | 85 | 90 | 90 | 89 | 79 |
| 8 | DHBT-3 | 100 | 84 | 78 | 74 | 80 | 80 | 76 | 78 | 82 | 85 | 80 | 54 | 81 | 83 | 82 | 85 |
| 9 | PB-1 | 100 | 91 | 69 | 70 | 80 | 77 | 81 | 82 | 70 | 72 | 77 | 64 | 85 | 86 | 89 | 84 |
| 10 | PB-6 | 100 | 70 | 60 | 59 | 72 | 75 | 80 | 84 | 81 | 73 | 66 | 61 | 77 | 82 | 75 | 62 |
| 11 | Other seeds and related entities | 284 | 100 | 99.65 | 98.59 | 100 | 100 | 100 | 99.65 | 98.94 | 98.59 | 100 | 98.94 | 99.3 | 100 | 100 | 99.65 |

**Table S10.2: External validation-set (original and augmented images) accuracies on paddy seeds variety prediction multiple augmentation model (251 epochs).**

| **Sr. No.** | **Variety / Other seeds and related entities** | **No. of images used for validation** | **Aug16** | **Aug17** | **Aug18** | **Aug19** | **Aug20** | **Aug21** | **Aug22** | **Aug23** | **Aug24** | **Aug25** | **Aug26** | **Aug27** | **Aug28** | **Aug29** |
| --- | --- | --- | --- | --- | --- | --- | --- | --- | --- | --- | --- | --- | --- | --- | --- | --- |
| 1 | 1121 | 100 | 64 | 59 | 68 | 70 | 61 | 69 | 71 | 76 | 74 | 64 | 79 | 79 | 49 | 81 |
| 2 | 1509 | 100 | 91 | 85 | 94 | 95 | 92 | 95 | 95 | 95 | 93 | 89 | 92 | 93 | 81 | 95 |
| 3 | 1637 | 100 | 83 | 58 | 84 | 85 | 79 | 83 | 84 | 83 | 71 | 80 | 77 | 77 | 40 | 60 |
| 4 | 1718 | 100 | 40 | 22 | 43 | 46 | 40 | 43 | 37 | 39 | 23 | 46 | 32 | 28 | 15 | 33 |
| 5 | 1728 | 100 | 80 | 76 | 86 | 84 | 88 | 84 | 82 | 90 | 87 | 86 | 75 | 78 | 57 | 61 |
| 6 | BAS-370 | 100 | 99 | 75 | 98 | 99 | 97 | 98 | 99 | 98 | 99 | 71 | 89 | 96 | 99 | 99 |
| 7 | CSR-30 | 100 | 92 | 84 | 91 | 93 | 89 | 92 | 93 | 93 | 90 | 80 | 78 | 83 | 85 | 74 |
| 8 | DHBT-3 | 100 | 85 | 68 | 86 | 86 | 83 | 86 | 86 | 83 | 71 | 60 | 64 | 66 | 77 | 81 |
| 9 | PB-1 | 100 | 82 | 86 | 86 | 91 | 81 | 85 | 88 | 89 | 87 | 84 | 93 | 92 | 81 | 76 |
| 10 | PB-6 | 100 | 80 | 80 | 80 | 78 | 76 | 80 | 79 | 79 | 82 | 75 | 77 | 79 | 73 | 73 |
| 11 | Other seeds and related entities | 284 | 100 | 100 | 100 | 100 | 100 | 100 | 100 | 100 | 100 | 100 | 100 | 99.65 | 99.65 | 100 |

**Table S11.1: External validation-set (original and augmented images) accuracies on paddy seeds variety prediction multiple augmentation model (502 epochs).**

| **Sr. No.** | **Variety / Other seeds and related entities** | **No. of images used for validation** | **Aug1** | **Aug2** | **Aug3** | **Aug4** | **Aug5** | **Aug6** | **Aug7** | **Aug8** | **Aug9** | **Aug10** | **Aug11** | **Aug12** | **Aug13** | **Aug14** | **Aug15** |
| --- | --- | --- | --- | --- | --- | --- | --- | --- | --- | --- | --- | --- | --- | --- | --- | --- | --- |
| 1 | 1121 | 100 | 67 | 64 | 65 | 50 | 68 | 68 | 71 | 69 | 66 | 75 | 57 | 79 | 76 | 75 | 67 |
| 2 | 1509 | 100 | 89 | 78 | 79 | 84 | 89 | 91 | 91 | 90 | 91 | 95 | 73 | 95 | 93 | 95 | 76 |
| 3 | 1637 | 100 | 86 | 74 | 77 | 76 | 86 | 85 | 72 | 69 | 72 | 57 | 68 | 86 | 91 | 86 | 77 |
| 4 | 1718 | 100 | 59 | 68 | 61 | 70 | 65 | 61 | 55 | 57 | 49 | 58 | 57 | 64 | 73 | 62 | 51 |
| 5 | 1728 | 100 | 60 | 51 | 56 | 69 | 71 | 75 | 62 | 65 | 70 | 44 | 56 | 74 | 72 | 71 | 51 |
| 6 | BAS-370 | 100 | 98 | 99 | 94 | 100 | 100 | 100 | 100 | 99 | 98 | 100 | 85 | 98 | 100 | 100 | 94 |
| 7 | CSR-30 | 100 | 70 | 63 | 62 | 60 | 68 | 63 | 59 | 58 | 63 | 60 | 46 | 65 | 67 | 72 | 64 |
| 8 | DHBT-3 | 100 | 97 | 94 | 96 | 99 | 98 | 98 | 99 | 99 | 99 | 95 | 77 | 99 | 99 | 99 | 95 |
| 9 | PB-1 | 100 | 86 | 90 | 90 | 78 | 80 | 86 | 86 | 80 | 80 | 82 | 75 | 88 | 93 | 90 | 79 |
| 10 | PB-6 | 100 | 58 | 47 | 56 | 54 | 58 | 60 | 66 | 63 | 60 | 58 | 51 | 63 | 66 | 67 | 54 |
| 11 | Other seeds and related entities | 284 | 99.3 | 99.65 | 99.3 | 100 | 100 | 98.94 | 98.94 | 98.24 | 99.3 | 99.65 | 99.65 | 99.3 | 100 | 99.65 | 98.94 |

**Table S11.2: External validation-set (original and augmented images) accuracies on paddy seeds variety prediction multiple augmentation model (502 epochs).**

| **Sr. No.** | **Variety / Other seeds and related entities** | **No. of images used for validation** | **Aug16** | **Aug17** | **Aug18** | **Aug19** | **Aug20** | **Aug21** | **Aug22** | **Aug23** | **Aug24** | **Aug25** | **Aug26** | **Aug27** | **Aug28** | **Aug29** |
| --- | --- | --- | --- | --- | --- | --- | --- | --- | --- | --- | --- | --- | --- | --- | --- | --- |
| 1 | 1121 | 100 | 69 | 61 | 73 | 79 | 81 | 78 | 77 | 76 | 72 | 66 | 78 | 77 | 60 | 72 |
| 2 | 1509 | 100 | 95 | 86 | 95 | 94 | 95 | 95 | 95 | 95 | 95 | 85 | 93 | 93 | 91 | 95 |
| 3 | 1637 | 100 | 87 | 80 | 90 | 87 | 91 | 91 | 92 | 91 | 85 | 84 | 86 | 85 | 58 | 69 |
| 4 | 1718 | 100 | 68 | 52 | 76 | 73 | 69 | 69 | 73 | 72 | 61 | 76 | 59 | 59 | 47 | 71 |
| 5 | 1728 | 100 | 65 | 63 | 72 | 65 | 65 | 73 | 71 | 65 | 73 | 71 | 68 | 69 | 54 | 49 |
| 6 | BAS-370 | 100 | 100 | 86 | 100 | 100 | 100 | 100 | 100 | 100 | 100 | 91 | 100 | 100 | 100 | 100 |
| 7 | CSR-30 | 100 | 75 | 55 | 77 | 80 | 69 | 79 | 77 | 78 | 63 | 51 | 59 | 61 | 57 | 60 |
| 8 | DHBT-3 | 100 | 99 | 98 | 99 | 99 | 99 | 99 | 99 | 99 | 97 | 92 | 91 | 94 | 96 | 99 |
| 9 | PB-1 | 100 | 90 | 88 | 92 | 91 | 90 | 89 | 87 | 94 | 88 | 80 | 94 | 91 | 84 | 91 |
| 10 | PB-6 | 100 | 66 | 61 | 67 | 64 | 66 | 66 | 66 | 65 | 65 | 63 | 64 | 64 | 67 | 64 |
| 11 | Other seeds and related entities | 284 | 100 | 99.3 | 100 | 99.65 | 99.65 | 100 | 100 | 100 | 100 | 99.65 | 100 | 100 | 100 | 100 |

**Table S12.1: External validation-set (augmented images only) accuracies on paddy seeds variety prediction multiple augmentation model (251 epochs).**

| **Sr. No.** | **Variety / Other seeds and related entities** | **No. of images used for validation** | **Aug1** | **Aug2** | **Aug3** | **Aug4** | **Aug5** | **Aug6** | **Aug7** | **Aug8** | **Aug9** | **Aug10** | **Aug11** | **Aug12** | **Aug13** | **Aug14** | **Aug15** |
| --- | --- | --- | --- | --- | --- | --- | --- | --- | --- | --- | --- | --- | --- | --- | --- | --- | --- |
| 1 | 1121 | 50 | 72 | 56 | 82 | 52 | 68 | 66 | 36 | 54 | 24 | 88 | 18 | 62 | 68 | 62 | 68 |
| 2 | 1509 | 50 | 78 | 76 | 60 | 88 | 78 | 78 | 68 | 94 | 90 | 98 | 58 | 96 | 94 | 94 | 36 |
| 3 | 1637 | 50 | 60 | 20 | 14 | 60 | 74 | 68 | 20 | 26 | 26 | 16 | 2 | 74 | 78 | 74 | 60 |
| 4 | 1718 | 50 | 24 | 18 | 4 | 38 | 44 | 28 | 14 | 30 | 18 | 28 | 18 | 38 | 62 | 44 | 98 |
| 5 | 1728 | 50 | 60 | 48 | 30 | 70 | 78 | 88 | 48 | 42 | 32 | 20 | 34 | 86 | 82 | 86 | 28 |
| 6 | BAS-370 | 50 | 94 | 82 | 82 | 92 | 94 | 94 | 100 | 100 | 100 | 100 | 66 | 86 | 96 | 70 | 82 |
| 7 | CSR-30 | 50 | 88 | 98 | 72 | 84 | 90 | 96 | 82 | 86 | 94 | 84 | 84 | 94 | 94 | 92 | 72 |
| 8 | DHBT-3 | 50 | 96 | 84 | 76 | 88 | 88 | 80 | 84 | 92 | 98 | 88 | 36 | 90 | 94 | 92 | 98 |
| 9 | PB-1 | 50 | 96 | 52 | 54 | 74 | 68 | 76 | 78 | 54 | 58 | 68 | 42 | 84 | 86 | 92 | 82 |
| 10 | PB-6 | 50 | 60 | 40 | 38 | 64 | 70 | 80 | 88 | 82 | 66 | 52 | 42 | 74 | 84 | 70 | 44 |
| 11 | Other seeds and related entities | 142 | 100 | 99.3 | 97.18 | 100 | 100 | 100 | 99.3 | 97.89 | 97.18 | 100 | 97.89 | 98.59 | 100 | 100 | 99.3 |

**Table S12.2: External validation-set (augmented images only) accuracies on paddy seeds variety prediction multiple augmentation model (251 epochs).**

| **Sr. No.** | **Variety / Other seeds and related entities** | **No. of images used for validation** | **Aug16** | **Aug17** | **Aug18** | **Aug19** | **Aug20** | **Aug21** | **Aug22** | **Aug23** | **Aug24** | **Aug25** | **Aug26** | **Aug27** | **Aug28** | **Aug29** |
| --- | --- | --- | --- | --- | --- | --- | --- | --- | --- | --- | --- | --- | --- | --- | --- | --- |
| 1 | 1121 | 50 | 54 | 44 | 62 | 66 | 48 | 64 | 68 | 78 | 74 | 54 | 84 | 84 | 24 | 88 |
| 2 | 1509 | 50 | 90 | 78 | 96 | 98 | 92 | 98 | 98 | 98 | 94 | 86 | 92 | 94 | 70 | 98 |
| 3 | 1637 | 50 | 90 | 40 | 92 | 94 | 82 | 90 | 92 | 90 | 66 | 84 | 78 | 78 | 4 | 44 |
| 4 | 1718 | 50 | 54 | 18 | 60 | 66 | 54 | 60 | 48 | 52 | 20 | 66 | 38 | 30 | 4 | 40 |
| 5 | 1728 | 50 | 74 | 66 | 86 | 82 | 90 | 82 | 78 | 94 | 88 | 86 | 64 | 70 | 28 | 36 |
| 6 | BAS-370 | 50 | 100 | 52 | 98 | 100 | 96 | 98 | 100 | 98 | 100 | 44 | 80 | 94 | 100 | 100 |
| 7 | CSR-30 | 50 | 98 | 82 | 96 | 100 | 92 | 98 | 100 | 100 | 94 | 74 | 70 | 80 | 84 | 62 |
| 8 | DHBT-3 | 50 | 98 | 64 | 100 | 100 | 94 | 100 | 100 | 94 | 70 | 48 | 56 | 60 | 82 | 90 |
| 9 | PB-1 | 50 | 78 | 86 | 86 | 96 | 76 | 84 | 90 | 92 | 88 | 82 | 100 | 98 | 76 | 66 |
| 10 | PB-6 | 50 | 80 | 80 | 80 | 76 | 72 | 80 | 78 | 78 | 84 | 70 | 74 | 78 | 66 | 66 |
| 11 | Other seeds and related entities | 142 | 100 | 100 | 100 | 100 | 100 | 100 | 100 | 100 | 100 | 100 | 100 | 99.3 | 99.3 | 100 |

**Table S13.1: External validation-set (augmented images only) accuracies on paddy seeds variety prediction multiple augmentation model (502 epochs).**

| **Sr. No.** | **Variety / Other seeds and related entities** | **No. of images used for validation** | **Aug1** | **Aug2** | **Aug3** | **Aug4** | **Aug5** | **Aug6** | **Aug7** | **Aug8** | **Aug9** | **Aug10** | **Aug11** | **Aug12** | **Aug13** | **Aug14** | **Aug15** |
| --- | --- | --- | --- | --- | --- | --- | --- | --- | --- | --- | --- | --- | --- | --- | --- | --- | --- |
| 1 | 1121 | 50 | 62 | 56 | 58 | 28 | 64 | 64 | 70 | 66 | 60 | 78 | 42 | 86 | 80 | 78 | 62 |
| 2 | 1509 | 50 | 86 | 64 | 66 | 76 | 86 | 90 | 90 | 88 | 90 | 98 | 54 | 98 | 94 | 98 | 60 |
| 3 | 1637 | 50 | 88 | 64 | 70 | 68 | 88 | 86 | 60 | 54 | 60 | 30 | 52 | 88 | 98 | 88 | 70 |
| 4 | 1718 | 50 | 56 | 74 | 60 | 78 | 68 | 60 | 48 | 52 | 36 | 54 | 52 | 66 | 84 | 62 | 40 |
| 5 | 1728 | 50 | 46 | 28 | 38 | 64 | 68 | 76 | 50 | 56 | 66 | 14 | 38 | 74 | 70 | 68 | 28 |
| 6 | BAS-370 | 50 | 96 | 98 | 88 | 100 | 100 | 100 | 100 | 98 | 96 | 100 | 70 | 96 | 100 | 100 | 88 |
| 7 | CSR-30 | 50 | 76 | 62 | 60 | 56 | 72 | 62 | 54 | 52 | 62 | 56 | 28 | 66 | 70 | 80 | 64 |
| 8 | DHBT-3 | 50 | 96 | 90 | 94 | 100 | 98 | 98 | 100 | 100 | 100 | 92 | 56 | 100 | 100 | 100 | 92 |
| 9 | PB-1 | 50 | 82 | 90 | 90 | 66 | 70 | 82 | 82 | 70 | 70 | 74 | 60 | 86 | 96 | 90 | 68 |
| 10 | PB-6 | 50 | 52 | 30 | 48 | 44 | 52 | 56 | 68 | 62 | 56 | 52 | 38 | 62 | 68 | 70 | 44 |
| 11 | Other seeds and related entities | 142 | 98.59 | 99.3 | 98.59 | 100 | 100 | 97.89 | 97.89 | 96.48 | 98.59 | 99.3 | 99.3 | 98.59 | 100 | 99.3 | 97.89 |

**Table S13.2: External validation-set (augmented images only) accuracies on paddy seeds variety prediction multiple augmentation model (502 epochs).**

| **Sr. No.** | **Variety / Other seeds and related entities** | **No. of images used for validation** | **Aug16** | **Aug17** | **Aug18** | **Aug19** | **Aug20** | **Aug21** | **Aug22** | **Aug23** | **Aug24** | **Aug25** | **Aug26** | **Aug27** | **Aug28** | **Aug29** |
| --- | --- | --- | --- | --- | --- | --- | --- | --- | --- | --- | --- | --- | --- | --- | --- | --- |
| 1 | 1121 | 50 | 66 | 50 | 74 | 86 | 90 | 84 | 82 | 80 | 72 | 60 | 84 | 82 | 48 | 72 |
| 2 | 1509 | 50 | 98 | 80 | 98 | 96 | 98 | 98 | 98 | 98 | 98 | 78 | 94 | 94 | 90 | 98 |
| 3 | 1637 | 50 | 90 | 76 | 96 | 90 | 98 | 98 | 100 | 98 | 86 | 84 | 88 | 86 | 32 | 54 |
| 4 | 1718 | 50 | 74 | 42 | 90 | 84 | 76 | 76 | 84 | 82 | 60 | 90 | 56 | 56 | 32 | 80 |
| 5 | 1728 | 50 | 56 | 52 | 70 | 56 | 56 | 72 | 68 | 56 | 72 | 68 | 62 | 64 | 34 | 24 |
| 6 | BAS-370 | 50 | 100 | 72 | 100 | 100 | 100 | 100 | 100 | 100 | 100 | 82 | 100 | 100 | 100 | 100 |
| 7 | CSR-30 | 50 | 86 | 46 | 90 | 96 | 74 | 94 | 90 | 92 | 62 | 38 | 54 | 58 | 50 | 56 |
| 8 | DHBT-3 | 50 | 100 | 98 | 100 | 100 | 100 | 100 | 100 | 100 | 96 | 86 | 84 | 90 | 94 | 100 |
| 9 | PB-1 | 50 | 90 | 86 | 94 | 92 | 90 | 88 | 84 | 98 | 86 | 70 | 98 | 92 | 78 | 92 |
| 10 | PB-6 | 50 | 68 | 58 | 70 | 64 | 68 | 68 | 68 | 66 | 66 | 62 | 64 | 64 | 70 | 64 |
| 11 | Other seeds and related entities | 142 | 100 | 98.59 | 100 | 99.3 | 99.3 | 100 | 100 | 100 | 100 | 99.3 | 100 | 100 | 100 | 100 |

**Supplementary Figures**

**Figure S1: Figure depicting the export statistics for basmati rice, from Pakistan. Source: REAP (Rice Export Association of Pakistan).**


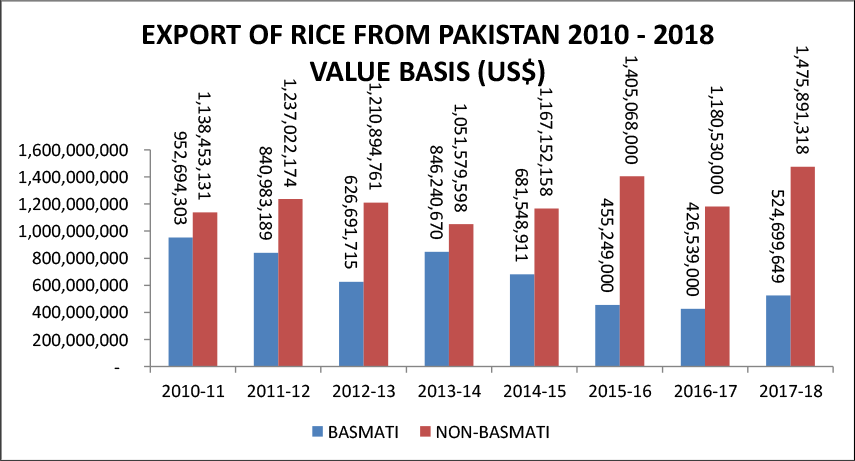


**Figure S2: Figure depicting the export statistics for basmati rice, top 30 countries are shown. Source: Data from APEDA, 2019.**

**
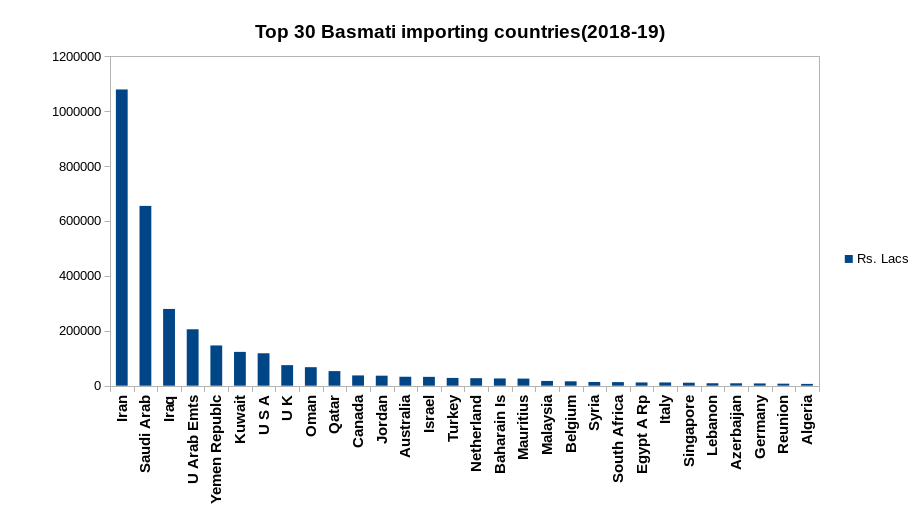
**

**Figure S3.1: Number of original images used in model generation.**


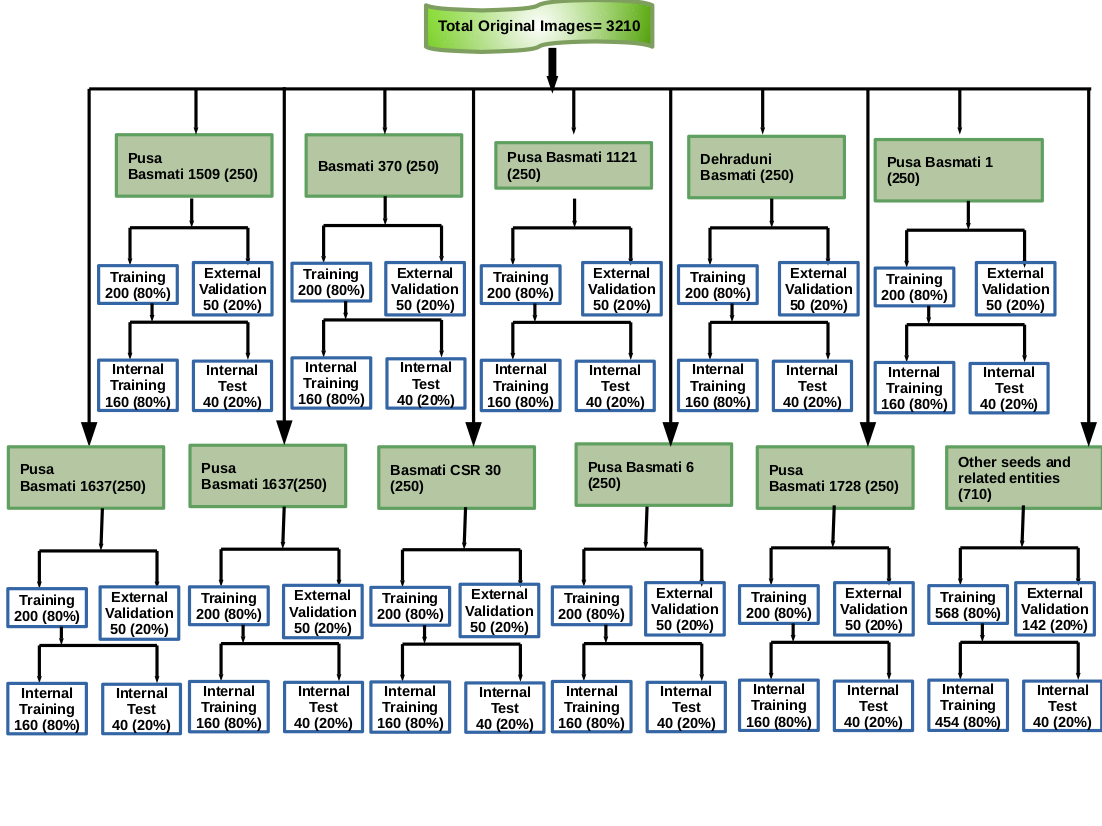


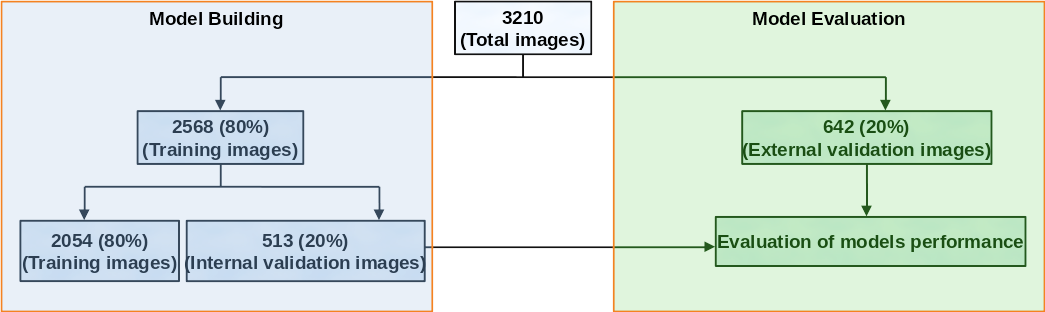
**Figure S3.2: Distribution of number of original images used in model generation.**

**Figure S4:**
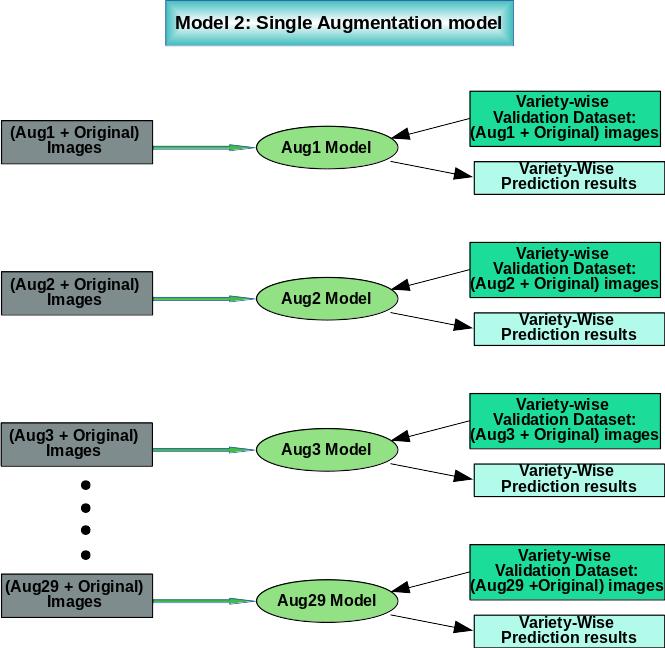
**Single augmentation and original images based prediction models (29 different types of models).**

**Figure S5:**
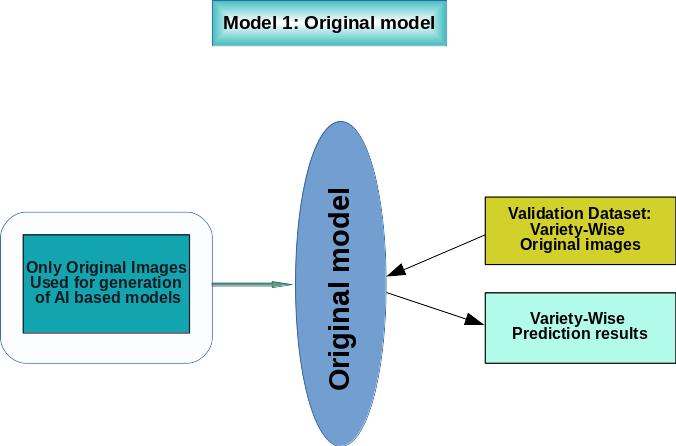
**For development of this model only original images were used. Validation dataset for this model also comprised of original images only.**

**Figure S6: Multiple augmentation model (251 and 502 epochs) tested on external validation set containing all augmentation types for each variety.**


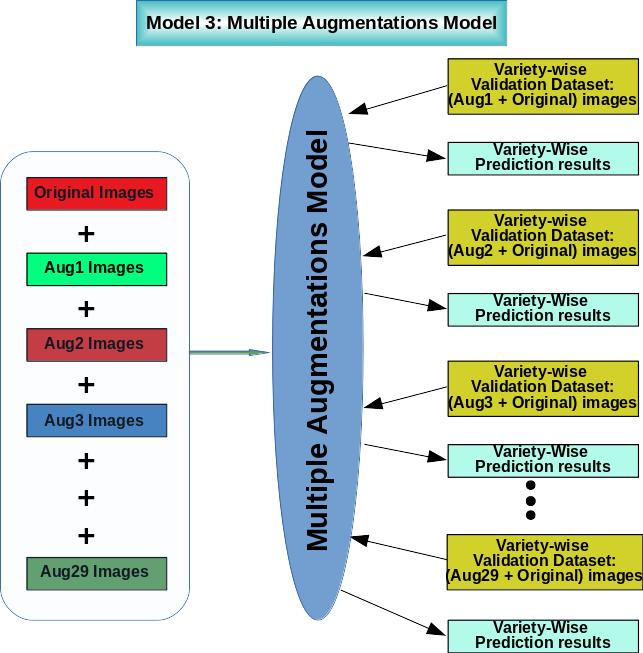


**Figure S7: Other seeds and related entities dataset preparation.**


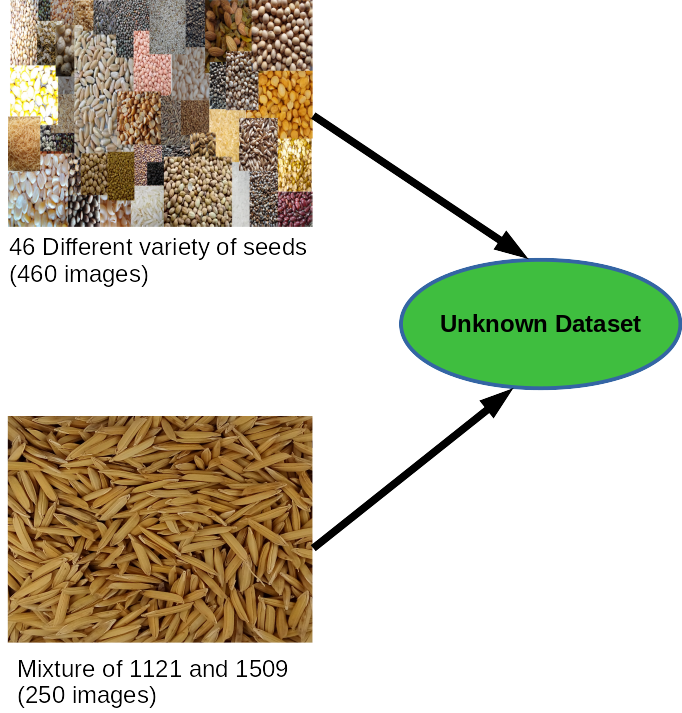


**Other seeds and related**

**entities dataset**

**Figure S8: Flow chart representing number of images used in model generation and as validation test set.**


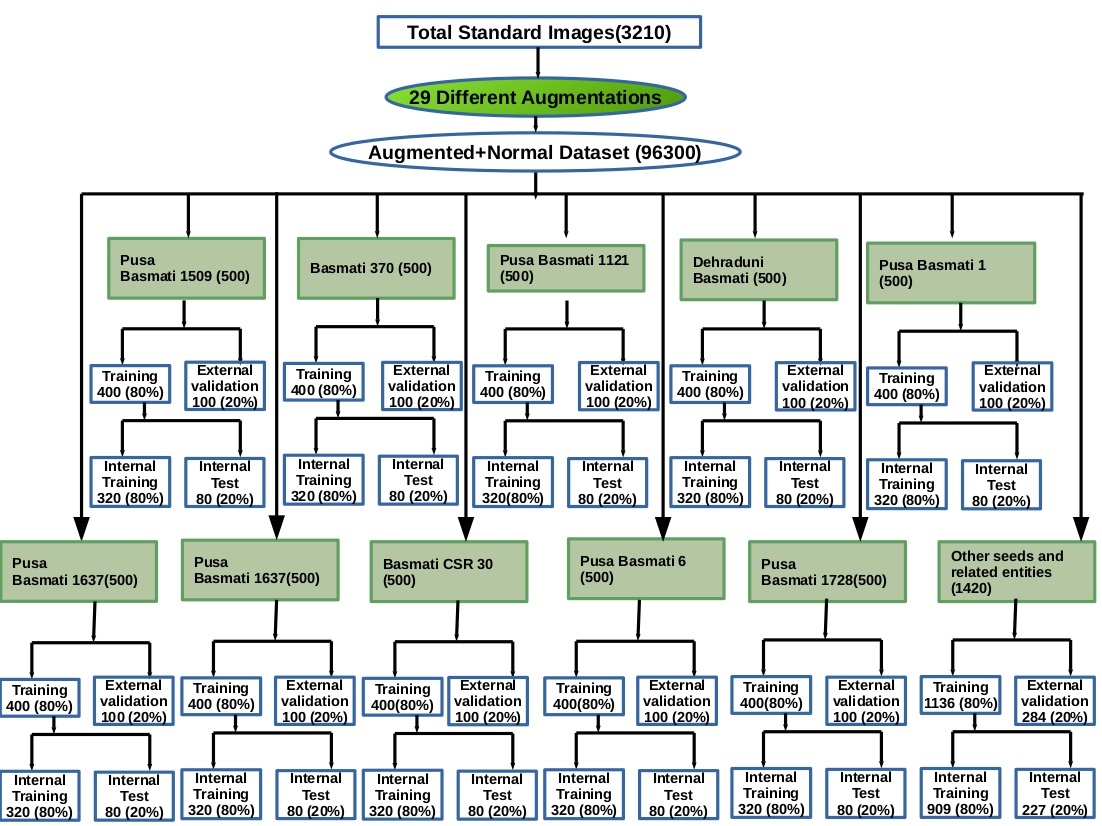

Supplement: Supplementary file 1 [file Table_1.docx]
